# Antibiotic prescribing and bacterial infection in COVID-19 inpatients in Southeast Asia: a systematic review and meta-analysis

**DOI:** 10.1093/jacamr/dlae093

**Published:** 2024-06-11

**Authors:** Achiraya Chanapal, Hung-Yung Cheng, Helen Lambert, Wenjuan Cong

**Affiliations:** Department of Population Health Sciences, Bristol Medical School, University of Bristol, 39 Whatley Road, Bristol BS8 2PS, UK; School of Medicine, University of Phayao, Phayao 56000, Thailand; Department of Population Health Sciences, Bristol Medical School, University of Bristol, 39 Whatley Road, Bristol BS8 2PS, UK; Department of Population Health Sciences, Bristol Medical School, University of Bristol, 39 Whatley Road, Bristol BS8 2PS, UK; Department of Population Health Sciences, Bristol Medical School, University of Bristol, 39 Whatley Road, Bristol BS8 2PS, UK

## Abstract

**Background:**

The prescribing of antibiotics to treat COVID-19 patients has been observed to occur frequently, often without clear justification. This trend raises concerns that it may have exacerbated antimicrobial resistance (AMR). Despite longstanding concerns over AMR in Southeast Asian countries, data on this issue are notably lacking.

**Objectives:**

To explore the impact of COVID-19 on antibiotic prescribing, bacterial infection prevalence and common resistant pathogens in COVID-19 inpatients.

**Methods:**

We searched PubMed, EMBASE, Web of Science and ThaiJO (a Thai academic database) to identify studies conducted in ASEAN member countries and published between December 2019 and March 2023. Screening and data extraction were done by two independent reviewers, with results synthesized using random-effects meta-analyses and descriptive statistical analyses. This review was registered with PROSPERO (CRD42023454337).

**Results:**

Of the 29 studies (19 750 confirmed COVID-19 cases) included for final analysis, the antibiotic prescribing rate was 62.0% (95%CI: 46.0%–76.0%) with a prescribing rate of 58.0% (21.0%–91.0%) in mild/moderate cases versus 91.0% (82.0%–98.0%) in severe/critical cases. Notably, 80.5% of antibiotics prescribed fall under the WHO AWaRe ‘Watch’ list, followed by ‘Access’ at 18.4% and ‘Reserve’ at 1.0%. The reported bacterial infection prevalence was 16.0% (7.0%–29.0%), with *Acinetobacter baumannii* being the most common resistant bacterium at 7.7%. Singapore was notable for its lower antibiotic prescribing rate of 17.0% and a lower bacterial infection rate of 10.0%.

**Conclusions:**

High antibiotic prescribing rates, disproportionate to bacterial infections and varying practices for COVID-19 inpatients across countries highlight the urgent need for this region to collaborate to tackle and mitigate AMR.

## Introduction

Antimicrobial resistance (AMR) is a significant global health threat, characterized by the loss of effectiveness of antimicrobial medicines in treating infections and leading to prolonged illnesses, extended hospital stays, increased healthcare costs, disability and death.^1^ Even before the COVID-19 pandemic, previous research suggested that nearly 5 million deaths globally in 2019 were related to AMR. Of these, around 1.3 million fatalities were directly caused by bacterial AMR.^2^ Similarly, O’Neill’s report projects up to 10 million annual deaths from AMR by 2050.^3^ The misuse and overuse of antimicrobials, particularly antibiotics, are primary drivers of AMR.^1,3^

The COVID-19 pandemic has further potentially intensified this concern, with numerous studies reporting an increase in antibiotic use among COVID-19 patients and a high prevalence of empiric antibiotic prescribing in hospitalized individuals.^4–6^ Our previous scoping review examining the initial phase of the pandemic (December 2019–June 2020) revealed that antibiotics were prescribed to 82.3% of COVID-19 patients worldwide, irrespective of the severity of illness.^4^ This is in accordance with a meta-analysis conducted during the same period, which found that about 75.0% of hospitalized COVID-19 patients received antibiotics, even though the estimated rate of bacterial co-infections was only 8.6%.^5^ These findings suggest that the likelihood of inappropriate or unjustified antibiotic use is high among COVID-19 patients. This trend could be explained by many factors including unknowns surrounding the virus, difficulties in rapidly excluding bacterial infections and the absence of treatment protocols during the initial stages of the pandemic.^4,7^ Moreover, individuals with severe or critical COVID-19 pneumonia tend to require mechanical ventilators and undergo multiple catheter insertions, potentially leading to ventilator-associated bacterial infections and catheter-related bloodstream infections. They also generally have longer hospital stays compared to patients with mild and moderate severity and patients with other acute infections. These aspects increase their susceptibility to hospital-acquired infections, particularly owing to the potential for either empiric antibiotic prescribing or unnecessary antibiotic use in COVID-19 patients.^8,9^ These, in turn, heighten the risk of AMR.

The Association of Southeast Asian Nations (ASEAN), which includes the countries of Brunei, Cambodia, Indonesia, Laos, Malaysia, Myanmar, the Philippines, Singapore, Thailand and Vietnam, is an intergovernmental organization that facilitates exchanges in economics, politics, sociocultural aspects and health.^10^ In the realm of public health, these nations share similar challenges.^11^ Even before the COVID-19 pandemic, AMR was one of the major public health problems in these countries, burdening them with a disproportionately high prevalence of AMR.^12^ During the pandemic, they also shared a similar pattern in the impacts of the first and second waves of pandemic, with the first wave being relatively mild, while the second wave was much more severe.^13^ The pandemic may have influenced the prevalence of antibiotic use and exacerbated the escalating threat of AMR, posing a grave challenge to Southeast Asian countries. However, despite the growing concern about AMR, there is a notable lack of clinical data on antibiotic prescribing and bacterial infections among COVID-19 patients from these countries.

The aim of this review is therefore to explore the potential impact of COVID-19 on antibiotic prescribing and AMR by investigating:

1. The prevalence and patterns of antibiotic prescribing for treating COVID-19 inpatients in ASEAN countries.
2. The prevalence of bacterial infection in COVID-19 inpatients in ASEAN countries.
3. The most commonly resistant pathogens in COVID-19 inpatients.

## Materials and methods

This review identified, synthesized and analysed data from studies that reported on the prescribing of antibiotics and bacterial infections in COVID-19 inpatients in ASEAN countries, and specifically published between December 2019 and March 2023. The review reported adheres to the guidelines of the Preferred Reporting of Systematic Reviews and Meta-Analyses (PRISMA) (Table S1, available as Supplementary data at *JAC-AMR* Online).^14^ Additionally, the protocol has been registered with PROSPERO (CRD42023454337).

### Search strategy

We conducted a search to identify relevant studies from the following databases: PubMed, EMBASE, Web of Science and one Thai academic database (ThaiJO). We included studies that were published in either English or Thai. Limits were set on the date of publication from 1 December 2019 to 31 March 2023. The search strategy can be found in Table S2.

### Inclusion and exclusion criteria and process for selecting studies

We included observational studies, such as cross-sectional, cohort (prospective or retrospective), case control, case series and controlled trials reporting either antibiotic prescribing or bacterial infection in COVID-19 patients conducted in ASEAN countries. Additionally, we included studies reporting on COVID-19 patients (confirmed by RT-PCR) in hospital settings, without restriction on age or gender. However, to ensure specificity to COVID-19 infection, we excluded cases with other respiratory viral co-infections. We also excluded reviews, editorials, case reports, trial protocols, clinical guidelines, case series with fewer than 10 patients, conference abstracts and qualitative studies and surveys.

During the first phase of screening, titles and abstracts obtained from electronic databases were screened by AC and WC, using EndNote and Microsoft Excel. The titles and abstracts that clearly did not meet the inclusion criteria and matched the exclusion criteria were excluded at this stage. Subsequently, full-text versions of all remaining references were examined in detail. In this second stage, any articles excluded were recorded, accompanied by the specific reasons for exclusion. A reviewer (AC) used Microsoft Excel to record reference details and decisions, which were then checked by a second reviewer (WC). Any discrepancies were resolved through consensus.

### Data extraction

To extract data, standardized data extraction forms were used on Microsoft Excel. These forms, modified from our published review,^15^ included publication details; study and patient characteristics; antibiotic prescribing rates for general COVID-19 inpatients, and for those with mild to moderate severity and severe to critical severity; reasons for antibiotic prescribing and bacterial infection rate for general COVID-19 inpatients [bacterial infection was defined as an infection with either documented confirmation or laboratory evidences (e.g. microbiological tests)]; co- and secondary bacterial infections (we defined co-infections as infections acquired in the community or confirmed within 48 h of hospital admission, and secondary infections as infections that developed after 48 h of hospitalization). In this review, we further extracted specific data including the number of prescriptions for each antibiotic therapy, and the number of patients with specific pathogens and resistant pathogens. Prior to full implementation, these forms underwent a pilot test on a limited set of papers and were adjusted as needed. To minimize bias and errors, one reviewer performed the data extraction and a second reviewer verified the extracted data. In the case of disagreements, resolution was achieved through discussion or by involving a third reviewer if required. Given that the aim of this review is to describe the prevalence and pattern of antibiotic treatment and the prevalence of bacterial infection among COVID-19 inpatients and not on the effects of a specific intervention, a comprehensive risk of bias or quality assessment was considered to be of lesser importance and was not undertaken.

### Data analysis and synthesis

We summarized the characteristics of the included studies, including patient types, study designs and study countries. We also analysed common scenarios of antibiotic prescribing, frequently prescribed antibiotics and frequently reported resistant pathogens among COVID-19 inpatients. To calculate the frequency of prescribed antibiotics, we examined the ratio of the number of prescriptions (including both monotherapy and combination regimen) for each antibiotic to the total number of antibiotic prescriptions. The proportion of resistant bacteria in COVID-19 inpatients was defined as the ratio of the number of patients with a specific resistant pathogen to the total number of patients on whose samples both bacterial culture and antimicrobial susceptibility or resistance were carried out. We further specified the AMR and multi-drug-resistant (MDR) rate among the samples infected with isolated organisms. These analyses were calculated using Microsoft Excel.

The prevalence of antibiotic prescribing or bacterial infection in each study was calculated as the percentage of patients who received antibiotics or had bacterial infections. For each percentage, we estimated 95% CIs using the exact method.^16^ If there were two or more studies, a meta-analysis was performed using Freeman–Tukey Double arcsine transformation and inverse variance meta-analysis approaches in random effects models via STATA Version 18.^17^ We also conducted subgroup meta-analyses for the antibiotic prescribing rate, categorized by the severity of patients (mild to moderate versus severe to critical) and study country. Additionally, we analysed bacterial infection rate by study country, co-infection and secondary infection. Heterogeneity was measured using the *I*^2^ statistic. For most outcomes, *I*^2^ values were high (>90%), so we complemented the meta-analysis results with descriptive statistics, accounting for each study's sample size in calculating pooled outcome estimates.

## Results

**Figure 1.**
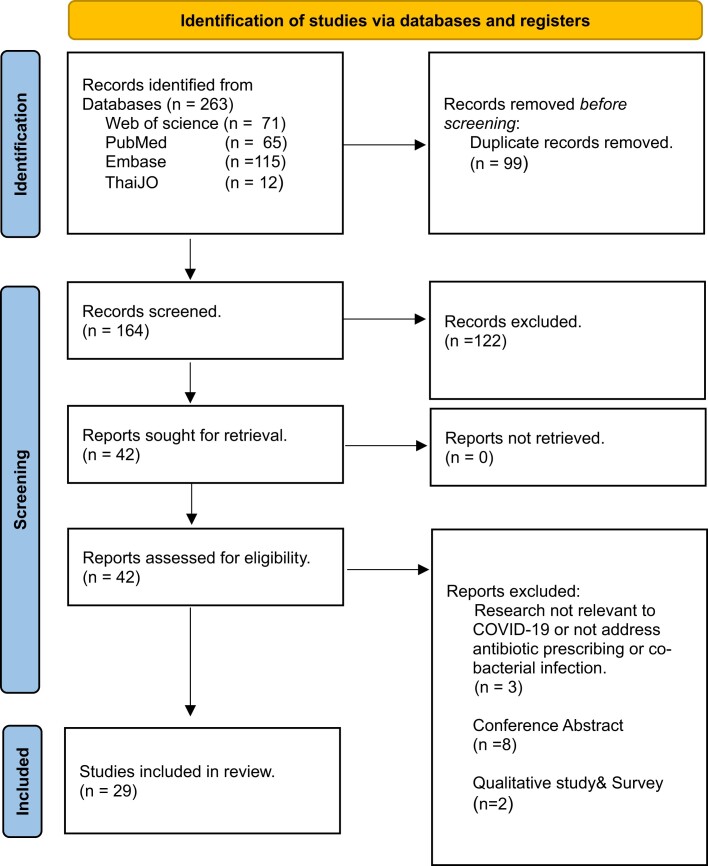
PRISMA 2020 flow diagram. *From:* Page *et al*.^14^ For more information, visit: http://www.prisma-statement.org/.

From a database search, a total of 263 records were identified. After removing duplicates and reviewing the abstracts and titles, 42 full records were screened for eligibility. Out of these, 13 additional records were excluded, including research not relevant to COVID-19 or not addressing antibiotic prescribing or co-bacterial infection, conference abstracts, and qualitative studies and surveys. Consequently, 29 studies involving 19 750 COVID-19 inpatients were included in the data analysis (see Figure 1 and Table S3). Among these, 24 studies reported on antibiotic prescribing and 17 studies focused on bacterial infection in COVID-19 patients, with 12 studies covering both topics. Regarding study design, most studies were cohort studies (*n* = 21), followed by cross-sectional studies (*n* = 6), randomized controlled trial (*n* = 1) and case series (*n* = 1). All studies were conducted from six countries in ASEAN, including Indonesia (*n* = 14), Malaysia (*n* = 5), The Philippines (*n* = 3), Singapore (*n* = 3), Thailand (*n* = 3) and Vietnam (*n* = 1). Notably, we did not have any studies from Brunei, Cambodia, Laos and Myanmar (Table 1).

**Table 1.** Summary of studies included in the meta-analysis

Total number of studies (patients)	29 (19 750)
Sample size^a^	247 (20–4043)
Percentage of males^a^	55.4% (43.9–55.4)^T^
Patient type	
Adults	27 (19 703)
Paediatric patients	2 (47)
Study design	
Cohort study	21 (11 855)
prospective	12 (7871)
retrospective	9 (3984)
Cross-sectional	6 (7351)
Randomize control trial	1 (500)
Case series	1 (44)
Country	
Indonesia	14 (8277)
Malaysia	5 (5153)
Philippines	3 (4040)
Singapore	3 (1495)
Thailand	3 (741)
Vietnam	1 (44)

^a^Median (range); ^T^k = 24 studies.

**Figure 2.**
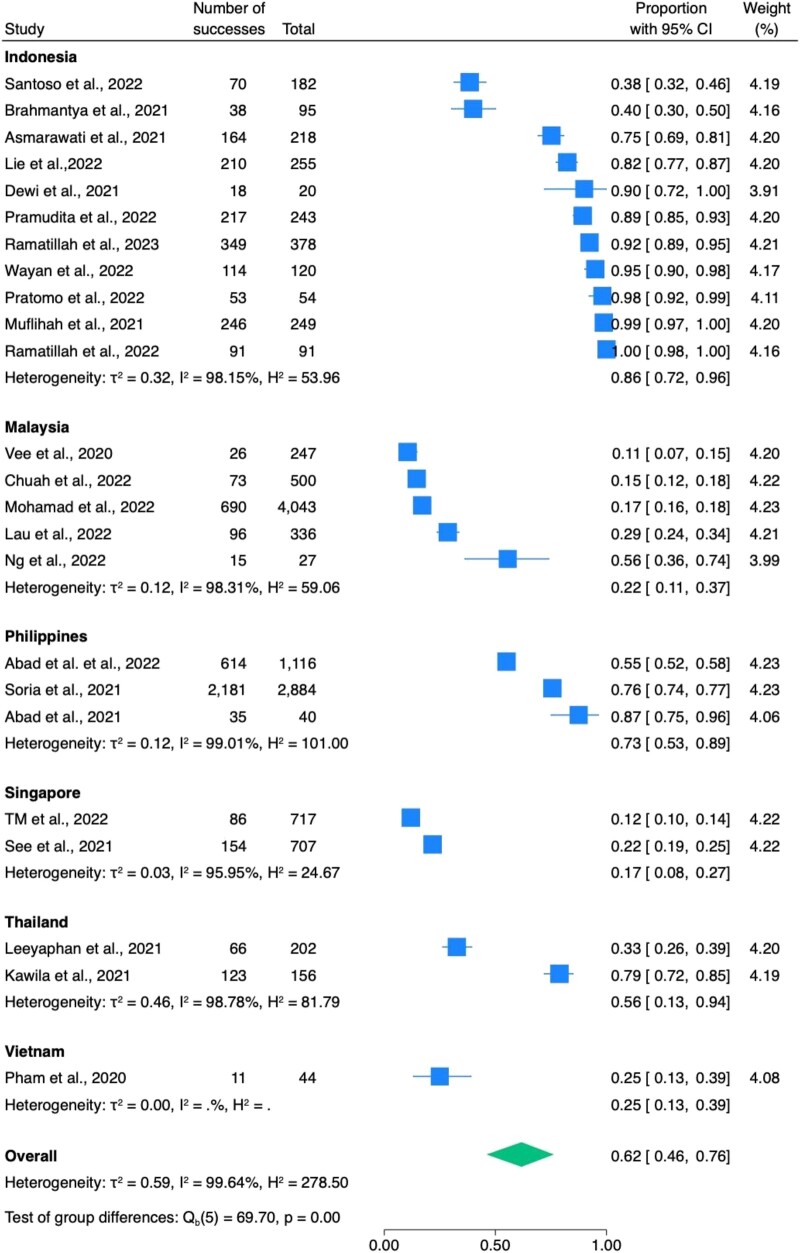
The prevalence of antibiotic prescribed in COVID-19 inpatients. H^2^ showed the proportion of variation due to heterogeneity, while τ^2^ estimated the variance of true effect sizes across studies.^19^

Antibiotics were prescribed to 5740 out of 12 924 inpatients with COVID-19. The overall antibiotic prescribing rate was 62.0% (95%CI: 46.0%–76.0%) (Table 2 and Figure 2). Among these, 1604 patients across seven studies were classified as having mild to moderate COVID-19, while 829 patients from nine studies were classified to have severe to critical COVID-19. Antibiotic prescribing rates were 58.0% (21.0%–91.0%) for mild to moderate cases (Figure S1A) and 91.0% (82.0%–98.0%), (*I*^2^ = 87.9%) for severe to critical cases (Figure S1B). Indonesia (*n* = 11) had the highest antibiotic prescribing rate at 86.0%, followed by the Philippines (*n* = 3) at 73.0%, Thailand (*n* = 2) at 56.0% and Vietnam (*n* = 1) at 25.0%. Meanwhile, Malaysia (*n* = 5) and Singapore (*n* = 2) had similarly lower rates at 22.0% and 17.0%, respectively (Figure 2). When we stratified antibiotic prescribing rates by economic status of the included countries based on the World Bank's classifications,^18^ we found that the lower-middle-income countries (Vietnam, the Philippines) had the highest rate of antibiotic prescribing at 69.6%. The upper-middle-income countries (Indonesia, Malaysia, Thailand) had the second-highest rate at 35.9%, followed by the high-income country (Singapore) at 16.9% (see Table S4).

**Table 2.** Prevalence of antibiotic prescribing in COVID-19 inpatients

	Studies (*n*)	Case/Total (*n*/*N*)	Prevalence (%)	
			Descriptive median (IQR)	Meta-analysis mean (95%CI)
Overall	24	5740/12 924	65.4 (27.7–89.5)	62.0% (46.0–76.0)
				I^2^ = 99.6%
Mild–moderate severity	7	466/1604	71.4 (22.4–91.9)	58.0% (21.0–91.0)
				I^2^ = 99.4%
Severe–critical severity	9	737/829	93.3 (88.9–98.1)	91.0 (82.0–98.0)
				I^2^ = 87.9%

Out of the 24 studies that reported on antibiotic prescribing in hospitalized COVID-19 cases, 15 provided reasons for antibiotic prescribing. These reasons were classified into two main scenarios during data extraction. The first scenario, ‘Confirmed Bacterial Infection’, referred to studies based on microbiological analysis. The second scenario, ‘Empirical Antibiotic Therapy’, was characterized by studies mentioning presumptive treatment for patients not yet confirmed through tests but suspected of having bacterial infections based on clinical presentation. This included prescribing antibiotics for solely suspected bacterial infections, suspected infections with pending microbiological test results and prescribing following recommended local guidelines. The majority of these studies (93.3%, *n* = 14), prescribed antibiotics based on the empirical antibiotic therapy scenario. This scenario included four studies citing suspected bacterial infections as the rationale for antibiotic prescribing, five studies citing recommended guidelines and five studies citing suspected bacterial infections with pending microbiological tests. Only one study (6.7%) prescribed antibiotics to COVID-19 inpatients due to confirmed bacterial infections (Table 3).

**Table 3.** Frequent antibiotic prescribing scenarios in COVID-19 patients

Antibiotic prescribing scenario	Reason for antibiotic prescribing for COVID-19 Patients	Number of studies reported	% of Total studies
Confirmed bacterial infection	Microbiological analysis of samples such as blood, stool, urine or sputum culture was conducted	1	6.7
Empirical antibiotic therapy	Empirical treatment for suspected bacterial infections	4	26.7
	Empirical antibiotics used as recommended national or international guidelines	5	33.3
	Empirical antibiotic prescription with some or all pending microbiological test confirmation for suspected bacterial infections	5	33.3

**Figure 3.**
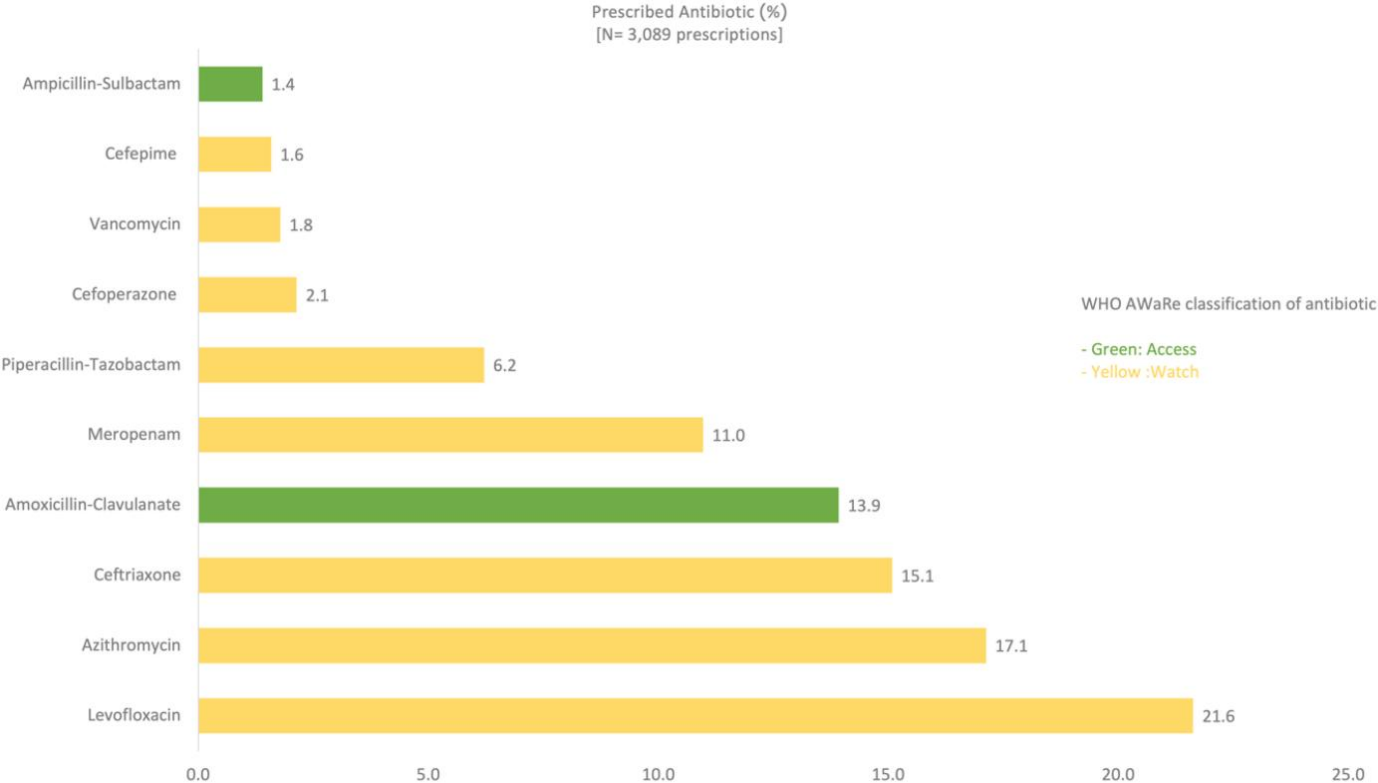
Top 10 most frequently prescribed antibiotics for COVID-19 inpatients.

Regarding frequently prescribed antibiotics, 10 studies, including a total of 6359 patients, provided detailed data on antibiotic prescriptions. We discovered that 1929 of these patients were prescribed antibiotics; however, some received monotherapy, while others were given a combination regimen consisting of more than one antibiotic, resulting in a total of 3089 antibiotic prescriptions issued (Table S5). The majority (80.5%) of the prescriptions fell under the WHO AWaRe Classification of Antibiotics ‘Watch’ list, followed by ‘Access’ at 18.4%, and ‘Reserve’ at 1% of the prescriptions. The top 10 most frequently prescribed antibiotics in ASEAN countries included eight from the ‘Watch’ category and two from the ‘Access’ category. The three most frequently prescribed antibiotics for COVID-19 inpatients were Levofloxacin (21.6%), Azithromycin (17.1%) and Ceftriaxone (15.1%), all of which are in the ‘Watch’ category (Figure 3).

**Figure 4.**
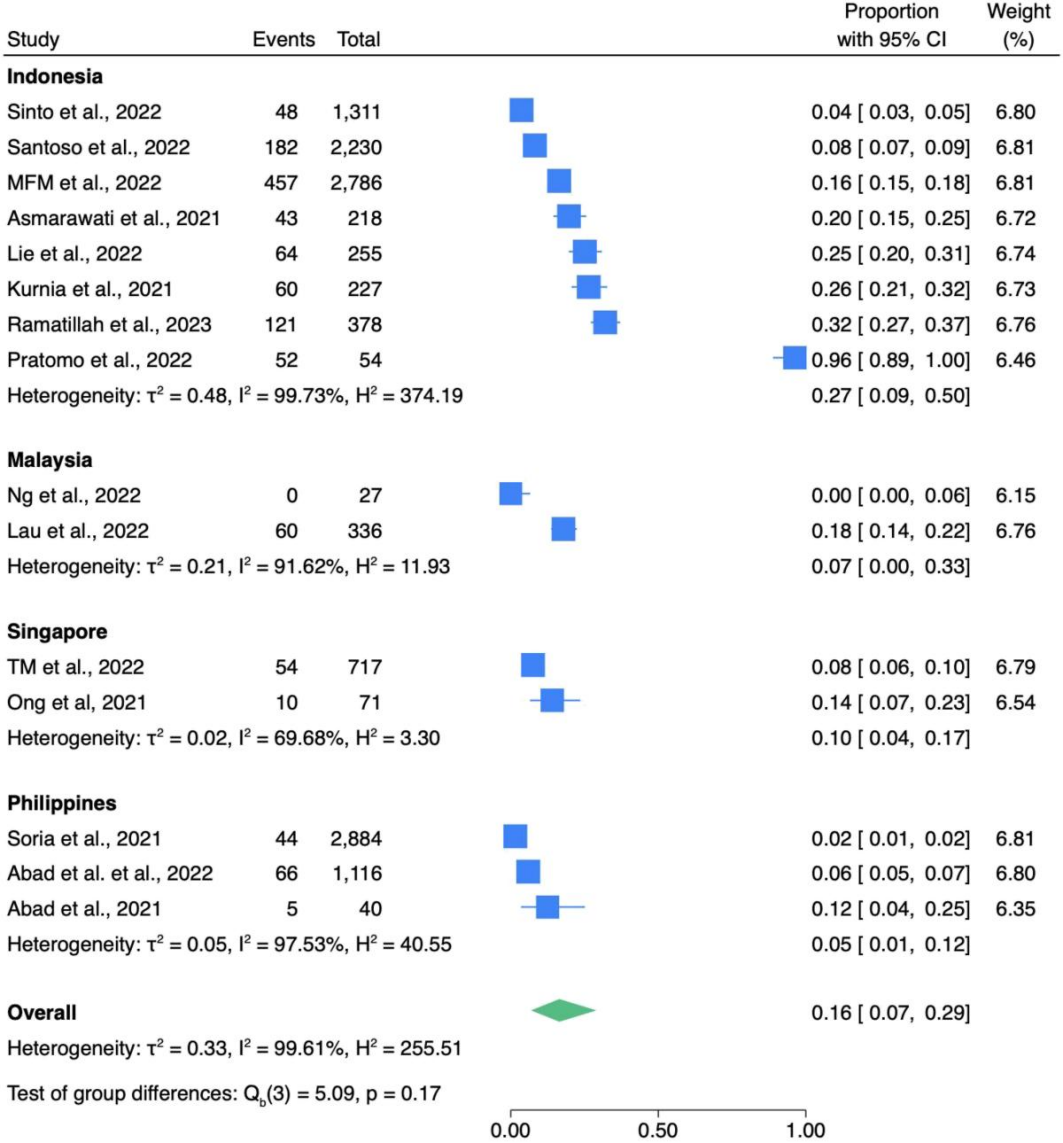
The prevalence of bacterial infection in COVID-19 inpatients. H^2^ showed the proportion of variation due to heterogeneity, while τ^2^ estimated the variance of true effect sizes across studies.^19^

Fifteen studies reported data on the number of hospitalized COVID-19 patients with bacterial infections. These studies included 1266 COVID-19 patients with bacterial infections among 12 650 COVID-19 patients and the overall bacterial infection rate was 16.0% (7.0%–29.0%) (Table 4 and Figure 4). Out of these, five studies with a total of 2642 COVID-19 patients reported 159 cases of co-infections, while seven studies involving 3204 patients reported 413 cases of secondary infections. Notably, secondary infections were more prevalent than co-infections, with rates of 26.0% (6.0%–52.0%) (Figure S1C) and 5.0% (2.0%–9.0%) (Figure S1D), respectively. Studies from only four countries (Indonesia, Malaysia, Philippines, Singapore) reported the number of bacterial infections in COVID-19 inpatients. Among these, those from Philippines (*n* = 3 studies) had the lowest rate of bacterial infection at 5.0% (1.0%–12.0%), followed by Malaysia (*n* = 2), Singapore (*n* = 2) and Indonesia (*n* = 8), with rates of 7.0% (0.0%–33.3%), 10.0% (4.0%–17.0%) and 27.0% (9.0%–50.0%) respectively (Figure 4). Regarding economic status, we found that studies from upper-middle-income countries (Indonesia, Malaysia) reported the highest bacterial infection rate in COVID-19 inpatients at 13.9% (13.1%–14.7%). Those from lower-middle-income countries (Philippines) had the lowest rate at 2.8% (2.3–3.4) (Table S6).

**Table 4.** Prevalence of bacterial infection in COVID-19 inpatients

	Studies(*n*)	Case/Total (*n*/*N*)	Prevalence (%)	
			Descriptive median (IQR)	Meta-analysis mean (95%CI)
Overall	15	1266/12 650	14.1 (6.7–22.4)	16.0% (7.0–29.0)
				I^2^ = 99.6%
Co-infection	5	159/2642	5.9 (4.6–7.5)	5.0 (2.0%–9.0%)
				I^2^ = 93.6%
Secondary infection	7	413/3204	14.5 (13.3–21.6)	26.0 (6.0–52.0)
				I^2^ = 99.3%

Among seven studies comprising 1181 COVID-19 patients that reported on resistant pathogens, we found that 18.0% of these cases involved resistant Gram-negative bacteria. The most frequently reported pathogen was *Acinetobacter baumannii* (7.7%) followed by *Pseudomonas aeruginosa* (3.3%), *Klebsiella pneumoniae* (3.2%) and *E. coli* (1.9%). Among the resistant Gram-positive bacteria, *Staphylococcus aureus* was the most prevalent, accounting for 0.5% as shown in Table 5. Additionally, for the samples from the four most common resistant organisms, we found that the AMR rates were 48.9%, 83.0%, 40.0% and 91.7%, respectively (Table S7). MDR rates were detected in *Acinetobacter baumannii* and *Pseudomonas aeruginosa* at rates of 43.0% and 68.1%, respectively (Table S8).

**Table 5.** Frequently reported resistant pathogens in COVID-19 patients

Pathogens	*N*	% among COVID-19 patients (1181)
Gram-negative bacteria	*n* = 212	18.0
*Acinetobacter baumannii*	91	7.7
*Pseudomonas aeruginosa*	39	3.3
*Klebsiella pneumoniae*	38	3.2
*E. coli*	22	1.9
*Stenotrophomonas maltophilia*	3	0.3
*Pantoea sp*	1	0.1
*Elizabethkingia meningospetica*	1	0.1
*Burkholderia cepacia*	1	0.1
Non-specified *ESBL*	16	1.4
Gram-positive bacteria	*n* = 11	0.9
*Staphylococcus aureus*	6	0.5
*Staphylococcus epidermidis*	3	0.3
*Staphylococcus hemolyticus (CoNS)*	2	0.2

## Discussion

In our systematic review and meta-analysis evaluating antibiotic prescribing prevalence among COVID-19 inpatients in ASEAN countries from December 2019 to March 2023, we found that there was extensive prescribing of antibiotics, disproportionate to the rate of bacterial infection in hospitalized patients.

Antibiotic prescribing rate for hospitalized COVID-19 patients in ASEAN countries was 62.0% (46.0%–76.0%), a decrease from 87.5% (47.8%–98.2%) in East and Southeast Asia (excluding China) reported in a meta-analysis from January 2019 to June 2020.^5^ Our lower prescribing rate may be attributed to the extended study duration from late 2019 to March 2023, covering various pandemic phases. Early in the COVID-19 pandemic, there was a surge in antibiotic usage, but this trend declined in later stages.^15^ As knowledge about appropriate clinical management for COVID-19 increased,^19–22^ WHO and national guidelines were revised. For instance, antibiotics are now recommended for moderate cases only with clinical evidence of bacterial infection,^23–25^ reflected in our lower average antibiotic prescribing rate of 58.0% for mild or moderate COVID-19 patients. In ASEAN countries, severe and critical patients had a markedly higher prescribing prevalence, reaching 91.0%, possibly due to increased risk of hospital-acquired infections.^8,9^ However, there were still variations in antibiotic prescribing practices for inpatients with mild and moderate severity of COVID-19 among ASEAN member countries, which could be influenced by differing guidelines and prescribing practices in each country. Our findings are in line with a recent WHO report, highlighting significant global variations in antibiotic use for mild or moderate COVID-19 cases from 2020 to 2023. This underscores the need for a systematic evidence review and collaboration to support the development of forthcoming guidelines on antibiotic use in COVID-19 patients.^26^

When assessing prescribed antibiotics based on WHO AWaRe classification, 80.5% fell under the ‘Watch’ category. This aligns with recent reviews during the second phase of the pandemic and a specific study in Sierra Leone in 2020–2021.^15,26,27^ These findings emphasize the urgent need to strengthen antibiotic stewardship programmes and update clinical guidelines to provide clear directions on which antibiotics to use for specific infections, particularly first line drugs to avoid development of drug resistance.

Interestingly, the included studies from Singapore recorded a lower antibiotic prescribing rate (17.0%) and a comparatively low bacterial infection rate (10.0%) than those from other ASEAN countries. Notably, it was reported that Singapore was able to maintain stability in both prevalence and the quality of antimicrobial prescribing during the COVID-19 pandemic.^28^ This stability may be attributed to the presence of a well-established multidisciplinary antimicrobial stewardship programme (AMS) that was already in place prior to the pandemic. Singapore launched its National Strategic Action Plan on AMR in 2017 and since then has consistently implemented AMS. These initiatives span a variety of areas, including the development of antimicrobial use surveillance, funding to enhance manpower and infrastructure in laboratory capacities, and efforts to improve physician diagnoses through education.^29–31^

Our study found that 16.0% of patients in our included studies had bacterial infection. Secondary infections were more common than co-infections, with rates of 26.0% and 5.0%, respectively. The co-infection rates align with our recent scoping review during the second phase of the pandemic, as well as with the systematic review and meta-analysis by Langford and colleagues.^15,32^ However, the overall bacterial infection and secondary infection rates were higher in our study compared to those studies. These may imply limited or deficient Infection Prevention and Control (IPC) measures in hospital settings, which are crucial risk factors for the development of secondary bacterial infection and AMR.

The lower bacterial infection rate in Singapore, in contrast to Indonesia and Malaysia, aligns with increased investments in IPC where resources are more abundant.^33^ However, the Philippines stands out as an outlier, displaying the lowest bacterial infection rate. This anomaly may be explained by effective IPC measures implemented in Philippines hospitals.^34^ Conversely, the high antibiotic prescribing rate in the Philippines can be attributed to limitations in laboratory infrastructure and capacity. These health system resource limitations might lead to antibiotic overprescription, as clinicians may resort to empirical treatment without adequate diagnostic support.^33–37^ Consistent with this explanation, our analysis also found a clear inverse correlation between national economic status and antibiotic prescribing rates, with the least wealthy (lower-middle-income) countries having the highest rate of antibiotic prescribing (69.6%), followed by upper-middle-income countries (Indonesia, Malaysia, Thailand) (35.9%) and the single high-income country (Singapore) having the lowest rate (16.9%).

Regarding the prevalence of bacterial resistance, this study highlighted the predominance of Gram-negative bacteria, with *Acinetobacter baumannii* being the most commonly identified species, followed by *Pseudomonas aeruginosa*, *Klebsiella pneumoniae* and *E.coli*. These pathogens are closely linked to nosocomial infections.^38^ Another notable observation is that the top four resistant pathogens identified in our study align with the leading pathogens contributing to the AMR burden in 2019, as detailed in the most comprehensive analysis of AMR impact to date.^2^ They are also recognized as priority pathogens by the WHO.^39^ Additionally, among the samples from the four most common resistant organisms, we found that AMR rates were high, ranging from 48.0% to 91.7%, with almost all samples of *Acinetobacter baumannii* and *Pseudomonas aeruginosa* being MDR. We cannot compare AMR rates before and after COVID-19 due to limitations in interpretation from a small sample size in our study and the lack of AMR surveillance reports in this area.^40,41^ Although the WHO Global Antimicrobial Resistance and Use Surveillance System (GLASS) is designed to progressively incorporate data from AMR surveillance in humans and the use of antimicrobial medicines, the majority of countries in the ASEAN were not reporting indicator data to GLASS before the pandemic.^41^

Collecting data on antibiotic use are essential for understanding the rise of AMR. Unfortunately, even before the era of COVID-19, obtaining reliable estimates across the ASEAN was extremely challenging. Our study offers the most current synthesis of available data on the impact of the entire duration of the COVID-19 pandemic on antibiotic prescribing and bacterial infection patterns among COVID-19 inpatients in this region, including individual countries. However, our study has limitations. First, despite an exhaustive search across multiple electronic databases for studies from ASEAN countries, our review did not include studies from Cambodia, Myanmar, Laos and Brunei. Notably, Cambodia, Myanmar and Laos have documented evidence of frequent inappropriate antibiotic prescribing for general patients.^42–44^ This may result from the language restriction or from under-representation of those countries in COVID-related health research. These may skew our results, potentially underestimating the true effects and not capturing the full scope of antibiotic prescribing and bacterial infection in ASEAN countries. Secondly, in terms of the meta-analysis results, our *I*^2^ values were high (>90%), but interpreting *I*^2^ statistics in meta-analyses of prevalence should be approached cautiously. High heterogeneity is often anticipated in prevalence estimates due to various factors, such as differences in the time and location of the included studies and genuine variations in prevalence across populations.^45^ Although we focused on COVID-19 inpatients (99.7% of whom were adults), variations persisted due to different study designs and outcome measures. Some studies included in this review were not designed to investigate antibiotic prescribing in COVID-19 patients.

In conclusion, our review found that COVID-19 led to excessive antibiotic prescribing for COVID-19 inpatients and undermined rational drug use during the pandemic. Additionally, we discovered a significantly high rate of AMR and MDR in samples from COVID-19 inpatients, suggesting that unnecessary antibiotic use may be one contributing factor. The most commonly identified organisms were WHO priority pathogens and the most commonly used antibiotics were from the Watch category, significantly contributing to the AMR burden. Finally, we found antibiotic prescribing rates to be inversely associated with national economic status, indicating the importance of well-resourced health systems in limiting unnecessary antibiotic use. These findings highlight the importance of addressing AMR in ASEAN countries and the urgent need for effective antimicrobial stewardship as a crucial solution. Enhanced cooperation and knowledge-sharing with support from ASEAN, as a regional organization, could help to reduce the substantial disparities in national health policies and resource capacities across member countries. Such collaboration could create strategies to address and mitigate AMR and build a strong network to combat future pandemics that could worsen AMR in the region.

## Supplementary Material

dlae093_Supplementary_Data

## Supplementary data

Figure S1 and Tables S1 to S8 are available as Supplementary data at *JAC-AMR* Online.
