# Supplementary material for: Antibiotic prescribing and bacterial infection in COVID-19 inpatients in Southeast Asia: a systematic review and meta-analysis
Source: JAC Antimicrob Resist. 2024 Jun 11;6(3):dlae093. doi: 10.1093/jacamr/dlae093 (PMC11166085; doi:10.1093/jacamr/dlae093)
Supplement: dlae093_Supplementary_Data [file dlae093_supplementary_data.docx]

Table of Contents

**Supplementary**

[Table S1. PRISMA 2020 Checklist 1](#_Toc164862569)

[Table S2. Search strategy 4](#_Toc164862570)

[Table S3. Characteristics of studies 6](#_Toc164862571)

[Figure S1. Forest plots 7](#_Toc164862572)

[Table S4. Prevalence of antibiotic prescribing in COVID-19 inpatients stratified by economic status 10](#_Toc164862573)

[Table S5. Frequently prescribed antibiotics for COVID-19 inpatients 11](#_Toc164862574)

[Table S6. Prevalence of bacterial infection in COVID-19 inpatients stratified by economic status. 12](#_Toc164862575)

[Table S7. Rate of samples showing antimicrobial resistance among the four most common resistant organisms. 12](#_Toc164862576)

[Table S8. Rate of samples infected with MDR among the four most common resistant organisms. 12](#_Toc164862577)

[References 13](#_Toc164862578)

# **Table S1. PRISMA 2020 Checklist**

| **Section and Topic** | **Item #** | **Checklist item** | **Location where item is reported** |
| --- | --- | --- | --- |
| **TITLE** | | |  |
| Title | 1 | Identify the report as a systematic review. | Page 1 |
| **ABSTRACT** | | |  |
| Abstract | 2 | See the PRISMA 2020 for Abstracts checklist. | Page 2 |
| **INTRODUCTION** | | |  |
| Rationale | 3 | Describe the rationale for the review in the context of existing knowledge. | Page 3-4 |
| Objectives | 4 | Provide an explicit statement of the objective(s) or question(s) the review addresses. | Page 4 |
| **METHODS** | | |  |
| Eligibility criteria | 5 | Specify the inclusion and exclusion criteria for the review and how studies were grouped for the syntheses. | Page 5 |
| Information sources | 6 | Specify all databases, registers, websites, organisations, reference lists and other sources searched or consulted to identify studies. Specify the date when each source was last searched or consulted. | Page 4-5 |
| Search strategy | 7 | Present the full search strategies for all databases, registers and websites, including any filters and limits used. | Table S2 |
| Selection process | 8 | Specify the methods used to decide whether a study met the inclusion criteria of the review, including how many reviewers screened each record and each report retrieved, whether they worked independently, and if applicable, details of automation tools used in the process. | Page 5 |
| Data collection process | 9 | Specify the methods used to collect data from reports, including how many reviewers collected data from each report, whether they worked independently, any processes for obtaining or confirming data from study investigators, and if applicable, details of automation tools used in the process. | Page 5-6 |
| Data items | 10a | List and define all outcomes for which data were sought. Specify whether all results that were compatible with each outcome domain in each study were sought (e.g. for all measures, time points, analyses), and if not, the methods used to decide which results to collect. | Page 5-6 |
|  | 10b | List and define all other variables for which data were sought (e.g. participant and intervention characteristics, funding sources). Describe any assumptions made about any missing or unclear information. | Page 5-6 |
| Study risk of bias assessment | 11 | Specify the methods used to assess risk of bias in the included studies, including details of the tool(s) used, how many reviewers assessed each study and whether they worked independently, and if applicable, details of automation tools used in the process. | NA |
| Effect measures | 12 | Specify for each outcome the effect measure(s) (e.g. risk ratio, mean difference) used in the synthesis or presentation of results. | NA |
| Synthesis methods | 13a | Describe the processes used to decide which studies were eligible for each synthesis (e.g. tabulating the study intervention characteristics and comparing against the planned groups for each synthesis (item #5)). | Page 6-7 |
|  | 13b | Describe any methods required to prepare the data for presentation or synthesis, such as handling of missing summary statistics, or data conversions. | Page 6-7 |
|  | 13c | Describe any methods used to tabulate or visually display results of individual studies and syntheses. | Page 6-7 |
|  | 13d | Describe any methods used to synthesize results and provide a rationale for the choice(s). If meta-analysis was performed, describe the model(s), method(s) to identify the presence and extent of statistical heterogeneity, and software package(s) used. | Page 6-7 |
|  | 13e | Describe any methods used to explore possible causes of heterogeneity among study results (e.g. subgroup analysis, meta-regression). | Page 6-7 |
|  | 13f | Describe any sensitivity analyses conducted to assess robustness of the synthesized results. | NA |
| Reporting bias assessment | 14 | Describe any methods used to assess risk of bias due to missing results in a synthesis (arising from reporting biases). | NA |
| Certainty assessment | 15 | Describe any methods used to assess certainty (or confidence) in the body of evidence for an outcome. | NA |
| **RESULTS** | | |  |
| Study selection | 16a | Describe the results of the search and selection process, from the number of records identified in the search to the number of studies included in the review, ideally using a flow diagram. | Page 7 |
|  | 16b | Cite studies that might appear to meet the inclusion criteria, but which were excluded, and explain why they were excluded. | Page 7, Figure 1 |
| Study characteristics | 17 | Cite each included study and present its characteristics. | Table S3 |
| Risk of bias in studies | 18 | Present assessments of risk of bias for each included study. | NA |
| Results of individual studies | 19 | For all outcomes, present, for each study: (a) summary statistics for each group (where appropriate) and (b) an effect estimate and its precision (e.g. confidence/credible interval), ideally using structured tables or plots. | Page 7-9 |
| Results of syntheses | 20a | For each synthesis, briefly summarise the characteristics and risk of bias among contributing studies. | Page 7-9 |
|  | 20b | Present results of all statistical syntheses conducted. If meta-analysis was done, present for each the summary estimate and its precision (e.g. confidence/credible interval) and measures of statistical heterogeneity. If comparing groups, describe the direction of the effect. | Page 7-9 |
|  | 20c | Present results of all investigations of possible causes of heterogeneity among study results. | Page 7-9 |
|  | 20d | Present results of all sensitivity analyses conducted to assess the robustness of the synthesized results. | NA |
| Reporting biases | 21 | Present assessments of risk of bias due to missing results (arising from reporting biases) for each synthesis assessed. | NA |
| Certainty of evidence | 22 | Present assessments of certainty (or confidence) in the body of evidence for each outcome assessed. | NA |
| **DISCUSSION** | | |  |
| Discussion | 23a | Provide a general interpretation of the results in the context of other evidence. | Page 10-12 |
|  | 23b | Discuss any limitations of the evidence included in the review. | Page 12 |
|  | 23c | Discuss any limitations of the review processes used. | Page 12 |
|  | 23d | Discuss implications of the results for practice, policy, and future research. | Page 13 |
| **OTHER INFORMATION** | | |  |
| Registration and protocol | 24a | Provide registration information for the review, including register name and registration number, or state that the review was not registered. | Page 4 |
|  | 24b | Indicate where the review protocol can be accessed, or state that a protocol was not prepared. | Page 4 PROSPERO(CRD42023454337). |
|  | 24c | Describe and explain any amendments to information provided at registration or in the protocol. | Page 4 |
| Support | 25 | Describe sources of financial or non-financial support for the review, and the role of the funders or sponsors in the review. | NA |
| Competing interests | 26 | Declare any competing interests of review authors. | NA |
| Availability of data, code and other materials | 27 | Report which of the following are publicly available and where they can be found: template data collection forms; data extracted from included studies; data used for all analyses; analytic code; any other materials used in the review. | NA |

*From:*  Page MJ, McKenzie JE, Bossuyt PM, Boutron I, Hoffmann TC, Mulrow CD, et al. The PRISMA 2020 statement: an updated guideline for reporting systematic reviews. BMJ 2021;372:n71. doi: 10.1136/bmj.n71

For more information, visit: <http://www.prisma-statement.org/>

# **Table S2. Search strategy**

| **Database** | **Search term** |
| --- | --- |
| PubMed | **Concept 1:** COVID-19 & Antibiotic prescription  All Fields = (COVID-19 or SARS-CoV-2 or Coronavirus disease 2019 or severe acute respiratory syndrome coronavirus-2) and (antimicrobial*)  OR  All Fields = (COVID-19 or SARS-CoV-2 or Coronavirus disease 2019 or severe acute respiratory syndrome coronavirus-2) and (antibiotic*)  **Concept 2:** South-east Asia countries [Asean summit]  Title/Abstract = (South east Asia countr*) or (South-east Asia*) or  (Brunei) or (Cambodia) or (Indonesia) or (Laos) or (Malaysia) or (Myanmar or Burma) or (Philippines) or (Singapore) or (Thailand) or (Vietnam) |
| Embase | **Concept 1:** COVID-19 & Antibiotic prescription  All Field = antibiotic* or antibacterial*  AND  All Field = severe acute respiratory syndrome coronavirus-2 or COVID-19 or SARS-CoV-2 or Coronavirus disease 2019  **Concept 2:** South-east Asia countries [ASEAN]  Title/Abstract = (“South east Asia countr* OR South-east Asia*” or (Brunei or Cambodia or Indonesia or Laos or Malaysia or Myanmar or Burma or Philippines or Singapore or Thailand or Vietnam)) |
| Web of Science | **Concept 1:** COVID-19 & Antibiotic prescription  **All Fields = (COVID-19 and antibiotic*) or (SARS-CoV-2 and antibiotic*) or (Coronavirus disease 2019 and antibiotic*) or (severe acute respiratory syndrome coronavirus-2 and antibiotic*)**  **OR**  **ALL Fields = (COVID-19 and antimicrobial*) or (SARS-CoV-2 and antimicrobial*) or (Coronavirus disease 2019 and antimicrobial*) or (severe acute respiratory syndrome coronavirus-2 and antimicrobial*)**  **Concept2:** South-east Asia countries [ASEAN Definition]  Title/Abstract **TS = (South east Asia countr*) or (South-east Asia*) or(Brunei) or (Cambodia) or (Indonesia) or (Laos) or (Malaysia) or (Myanmar or Burma) or (the Philippines or Philippines) or (Singapore) or (Thailand) or (Vietnam)** |
| THAIJO | (โคโรนาไวรัส 2019 or ไวรัสโคโรนา 2019 or โควิด-19) and (ยาปฏิชีวนะ or ยาฆ่าเชื้อ) |

# **Table S3. Characteristics of studies**

| Study | Design | Study period | Country | Population size | % Male |
| --- | --- | --- | --- | --- | --- |
|  |  |  |  |  |  |
| Subagdja et al. | Cross-sectional | 03/2020 - 10/2021 | Indonesia | 2,786 | NA |
| Ng et al. | Cohort | 22/01/2020- 15/04/2020 | Singapore | 717 | 57.2 |
| Abad et al. | Cohort | 12/03/2020 - 31/08/2020 | Philippines | 1,116 | 52.5 |
| Sinto et al. | Cohort | 1/01/2019- 31/12/2020 | Indonesia | 1,311 | NA |
| Pratomo et al. | Cohort | 05/2020- 05/2021 | Indonesia | 54 | 61.1 |
| Pramudita et al. | Cohort | 20/3/2020 -31/7/2020 | Indonesia | 243 | NA |
| Muflihah et al. | Cohort | 07/2020 - 03/2021 | Indonesia | 249 | 55.4 |
| Ng et al. | Cohort | 1/04/ 2021 -31/10/ 2021. | Malaysia | 27 | 51.9 |
| Kurnia et al. | Cohort | 2020-2021 | Indonesia | 227 | 54.6 |
| Brahmantya et al. | Cross-sectional | 04/2020 - 05/2020 | Indonesia | 95 | 72.6 |
| Abad et al. | Cohort | 5/03/2020 -28/03 2020 | Philippines | 40 | 57.5 |
| Chuah et al. | A Randomized Control trial | 1/02/2021-20/07/2021 | Malaysia | 500 | 48.4 |
| Ramatillah et al. | Cohort | 03/2020 - 9/2021 | Indonesia | 378 | 47.9 |
| Leeyaphan et al. | Cohort | 01/2020 - 04/2020 | Thailand | 202 | 58.4 |
| Ramatillah et al. | Cohort | 03/2020 - 09/2020 | Indonesia | 91 | 70.3 |
| Mohamad et al. | Cross-sectional | 02/2020 - 04/2020 | Malaysia | 4,043 | 65.0 |
| Santoso et al. | Cohort | 03/2020 - 03/2021 | Indonesia | 2,230 | NA |
| Dewi et al. | Cross-sectional | 03/2020 - 10/2020 | Indonesia | 20 | 50.0 |
| Wayan et al. | Cohort | 04/2020 - 12/2020 | Indonesia | 120 | 83.3 |
| Ong et al. | Cross-sectional | 1/02/2020 - 30/06/ 2020 | Singapore | 71 | 83.1 |
| Lie et al. | Cohort | 03/2020 - 09/2020 | Indonesia | 255 | 52.2 |
| Soria et al. | Cohort | 1/02/2020 - 15/07/ 2020 | Philippines | 2,884 | 54.4 |
| Lau et al. | Cross-sectional | 2021 | Malaysia | 336 | NA |
| See et al. | Cohort | 23/01/2020 - 15 /04/ 2020 | Singapore | 707 | 57.0 |
| Vee et al. | Cohort | 09/3 - 1/04 2020 | Malaysia | 247 | 69.6 |
| Asmarawati et al. | Cohort | 14/03/2020 - 30/09/ 2020 | Indonesia | 218 | 55.1 |
| Pham et al. | Case series | 6/03/2020 - 15/04/2020 | Vietnam | 44 | 52.0 |
| Wannacharoen et al. | Cohort study | 1/07/2021 - 31/11/2021 | Thailand | 383 | 43.9 |
| Kawila et al. | Cohort study | 04/2021 - 10/2021 | Thailand | 156 | 65.0 |

# **Figure S1. Forest plots**


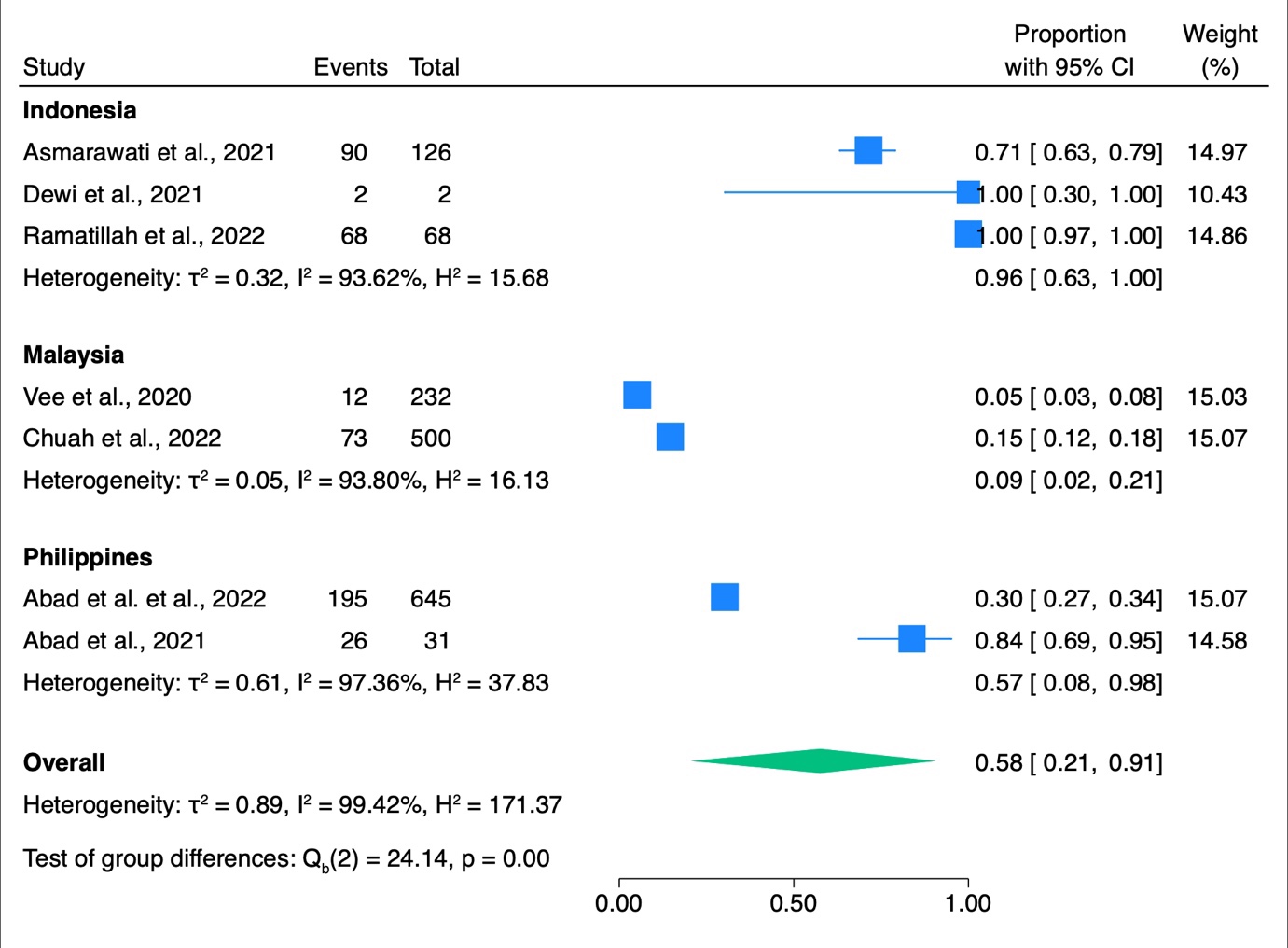


**Figure S1A. The prevalence of antibiotic prescribed in mild to moderate COVID-19 inpatients.**

**Figure S1B. The prevalence of antibiotic prescribed in severe to critical COVID-19 inpatients.**


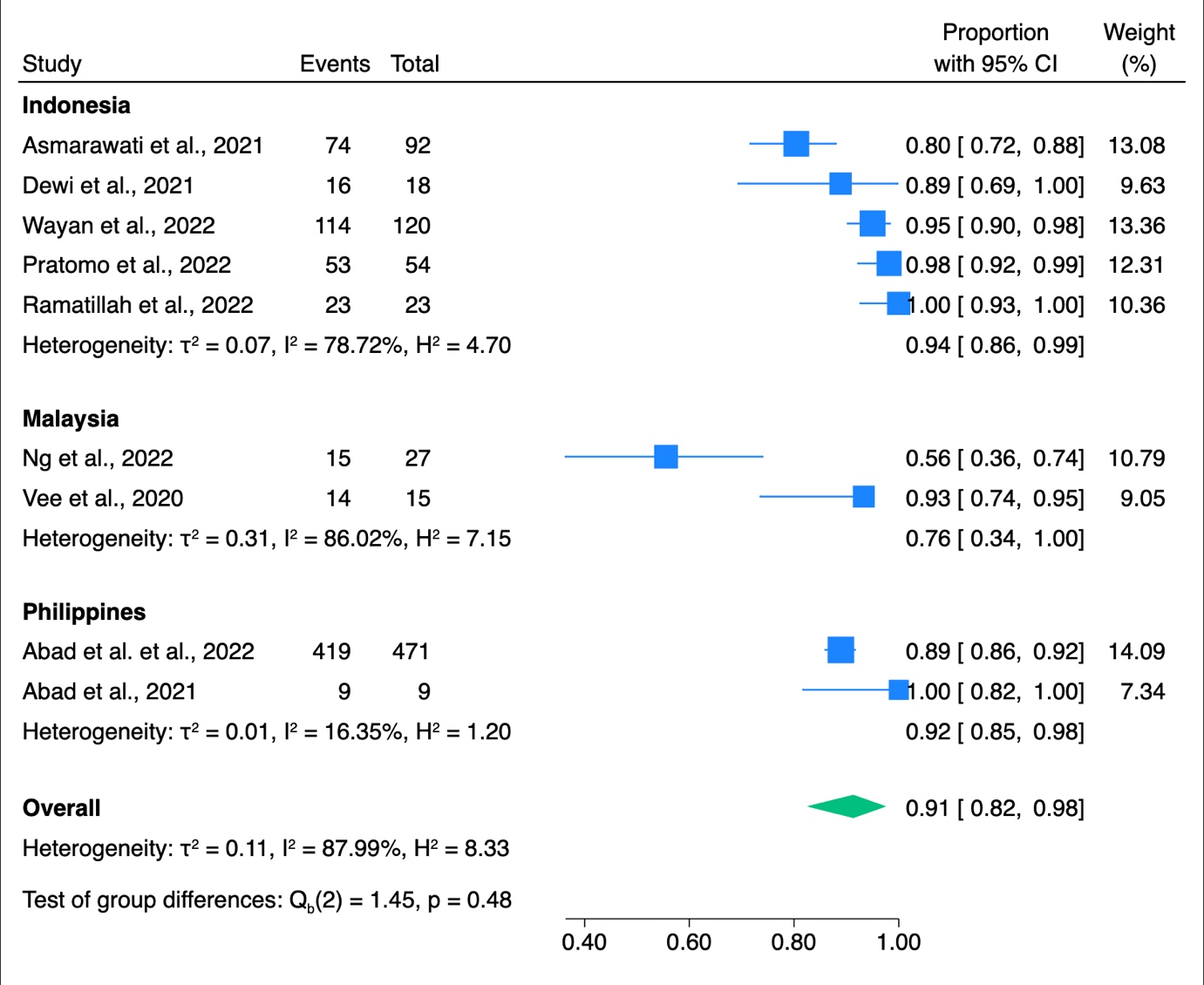

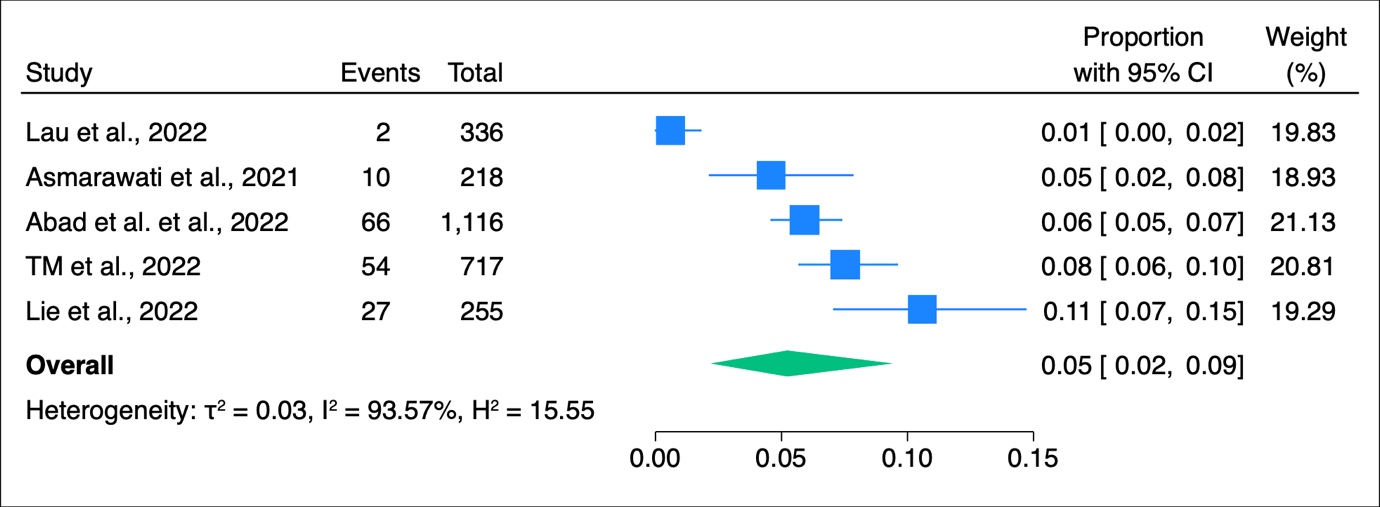


**Figure S1D The prevalence of co-bacterial infection in COVID-19 inpatients**


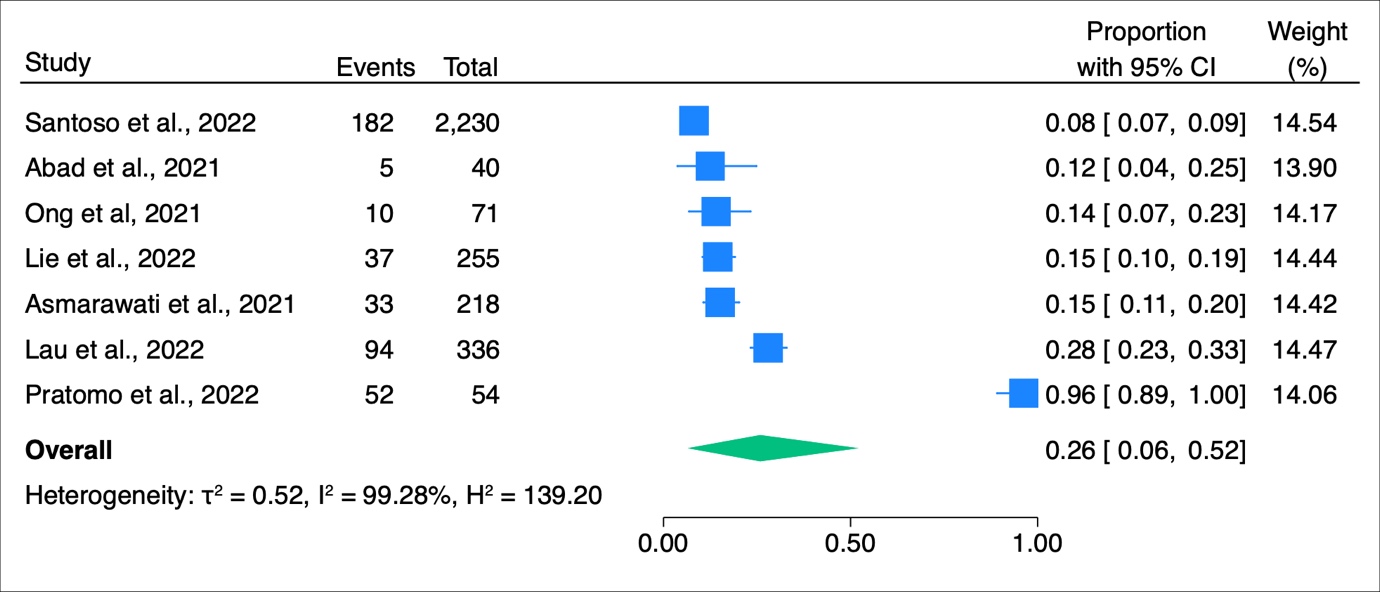


**Figure S1C. The prevalence of secondary bacterial infection in COVID-19 inpatients**

# **Table S4. Prevalence of antibiotic prescribing in COVID-19 inpatients stratified by economic status**

| Country | Antibiotic prescribing Prevalence (%) | | | |
| --- | --- | --- | --- | --- |
|  | N Study | Cases | Total | Mean (95%CI) |
| High income | 2 | 240 | 1,424 | 16.9 (14.9-18.8) |
| Upper-middle income | 10 | 2,659 | 7,416 | 35.9 (34.8-36.9) |
| Lower-middle income | 3 | 2,841 | 4,084 | 69.6 (68.2-71.0) |

# **Table S5.** **Frequently prescribed antibiotics for COVID-19 inpatients**

| **Antibiotic** | **AWaRe Classification** | **n**  **(total = 3,089)** | **Percentage of prescriptions (%)** |
| --- | --- | --- | --- |
| Levofloxacin | Watch | 668 | 21.6 |
| Azithromycin | Watch | 529 | 17.1 |
| Ceftriaxone | Watch | 466 | 15.1 |
| Amoxicillin-clavulanate | Access | 430 | 13.9 |
| Meropenam | Watch | 339 | 11.0 |
| Piperacillin-tazobactam | Watch | 192 | 6.2 |
| Cefoperazone | Watch | 66 | 2.1 |
| Vancomycin | Watch | 55 | 1.8 |
| Cefepime | Watch | 49 | 1.6 |
| Ampicillin-Sulbactam | Access | 43 | 1.4 |
| Ceftazidime | Watch | 33 | 1.1 |
| Colistin/Polymyxin B | Reserve | 29 | 0.9 |
| Moxifloxacin | Watch | 28 | 0.9 |
| Cefotaxime | Watch | 27 | 0.9 |
| Trimethoprim-sulfamethoxazole | Access | 20 | 0.7 |
| Cloxacillin | Access | 19 | 0.6 |
| Doxycycline | Access | 19 | 0.6 |
| Cefuroxime | Watch | 18 | 0.6 |
| Clindamycin | Access | 14 | 0.5 |
| Amikacin | Access | 11 | 0.4 |
| Amoxicillin | Access | 7 | 0.2 |
| Cefixime | Watch | 5 | 0.2 |
| Ciprofloxacin | Watch | 5 | 0.2 |
| Imipenam | Watch | 4 | 0.1 |
| Erythromycin | Watch | 3 | 0.1 |
| Cefazolin | Access | 2 | 0.1 |
| Cepalexin | Access | 2 | 0.1 |
| Fosfomycin [oral] | Watch | 2 | 0.1 |
| Gentamycin | Access | 1 | < 0.1 |
| Ertapenam | Watch | 1 | < 0.1 |
| Doripenem | Watch | 1 | < 0.1 |
| Cefoperazone sulbacatam | Watch | 1 | < 0.1 |

# **Table S6. Prevalence of bacterial infection in COVID-19 inpatients stratified by economic status.**

| Economic status | Bacterial infection Prevalence (%) | | | |
| --- | --- | --- | --- | --- |
|  | N Study | Cases | Total | Mean (95%CI) |
| High income | 2 | 64 | 788 | 8.1% (6.2-10.0). |
| Upper-middle income | 10 | 1,087 | 7,822 | 13.9% (13.1-14.7) |
| Lower-middle income | 3 | 115 | 4,040 | 2.8% (2.3-3.4) |

# **Table S7. Rate of samples showing antimicrobial resistance among the four most common resistant organisms.**

| **Bacteria** | **Number of samples infected with antibiotic-resistant organisms** | **Number of samples infected with isolated organisms** | **Rate of samples showing antimicrobial resistance (%)** |
| --- | --- | --- | --- |
| *Acinetobacter baumannii* | 91 | 186 | 48.9 |
| *Pseudomonas aeruginosa* | 39 | 47 | 83.0 |
| *Klebsiella pneumoniae* | 40 | 100 | 40.0 |
| *E.coli* | 22 | 24 | 91.7 |

# **Table S8. Rate of samples infected with MDR among the four most common resistant organisms.**

| **Bacteria** | **Number of samples infected with MDR organisms** | **Number of samples infected with isolated organisms** | **Rate of samples infected with MDR (%)** |
| --- | --- | --- | --- |
| *Acinetobacter baumannii* | 80 | 186 | 43.0 |
| *Pseudomonas aeruginosa* | 32 | 47 | 68.1 |

# **References**

1. Subagdja MFM, Sugianli AK, Prodjosoewojo S *et al.* Antibiotic Resistance in COVID-19 with Bacterial Infection: Laboratory-Based Surveillance Study at Single Tertiary Hospital in Indonesia. *Infection and Drug Resistance* 2022; **15**: 5849-56. <https://doi.org/10.2147/idr.S379324>
2. Ng TM, Ong SWX, Loo AYX *et al.* Antibiotic Therapy in the Treatment of COVID-19 Pneumonia: Who and When?. *Antibiotics* 2022; **11**(2). <https://doi.org/10.3390/antibiotics11020184>
3. Abad CL, Sandejas JCM, Poblete JB *et al.* Bacterial coinfection and antimicrobial use among patients with COVID-19 infection in a referral center in the Philippines: A retrospective cohort study. *IJID Reg* 2022; **4**: 123-30. <https://doi.org/10.1016/j.ijregi.2022.07.003>
4. Sinto R, Lie KC, Setiati S *et al.* Blood culture utilization and epidemiology of antimicrobial-resistant bloodstream infections before and during the COVID-19 pandemic in the Indonesian national referral hospital. *Antimicrobial Resistance and Infection Control* 2022; **11**(1):12. <https://doi.org/10.1186/s13756-022-01114-x>
5. Pratomo IP, Priyonugroho G, Baskoro H *et al.* Bronchoscopy Findings of Severe and Critical COVID-19 Patients Treated in ICU: A Year of Experience in a Developing Country. *Open Respiratory Medicine Journal* 2022; **16**(1) [https://doi.org/](https://doi.org/10.3390/antibiotics11020184)[10.2174/18743064-v16-e221020-2022-11](https://doi.org/10.2174%2F18743064-v16-e221020-2022-11)
6. Pramudita A, Rosidah S, Yudia N *et al.* Cardiometabolic Morbidity and Other Prognostic Factors for Mortality in Adult Hospitalized COVID-19 Patients in North Jakarta, Indonesia. *Global Heart* 2022;**17**(1) <https://doi.org/10.5334/GH.1019>
7. Muflihah H, Bhekti Rahimah S, Widiyanto T *et al*. Clinical use of antiviral, antibiotic and immunomodulatory drugs in hospitalized COVID-19 patients: a retrospective study in Bandung, Indonesia. *F1000Research* 2021; **10**. [https://doi.org/10.12688/f1000research.73606.1](https://doi.org/https://dx.doi.org/10.12688/f1000research.73606.1)
8. Ng DCE, Tan KK, Ting GSS *et al.* Comparison of Severe Viral Pneumonia Caused by SARS-CoV-2 and Other Respiratory Viruses Among Malaysian Children During the COVID-19 Pandemic. *Frontiers in Pediatrics* 2022;**10**. <https://doi.org/10.3389/fped.2022.865099>.
9. Kurnia D. Correlation between Multidrug Resistance Infection with Clinical Outcomes of Critically ill Patients with COVID-19 Admitted to an Intensive Care at RSUP Dr. M. Djamil in Indonesia. *Open Access Macedonian Journal of Medical Sciences* 2022; **10**(B): 972-7. <https://doi.org/10.3889/oamjms.2022.9105>
10. Brahmantya IBY, Purnamasidhi CAW, Sumardika IW. COVID-19 pharmacological treatment at the Udayana University Hospital in April-May 2020. *Biomedical and Pharmacology Journal* 2021; **14**(2): 971-7. <https://doi.org/10.13005/bpj/2198>
11. Abad CL, Lansang MAD, Cordero CP *et al.* Early experience with COVID-19 patients in a private tertiary hospital in the Philippines: Implications on surge capacity, healthcare systems response, and clinical care. *Clinical Epidemiology and Global Health* 2021;**10**. <https://doi.org/10.1016/j.cegh.2020.100695>
12. Chuah CH, Chow TS, Hor CP *et al.* Efficacy of Early Treatment with Favipiravir on Disease Progression among High-Risk Patients with Coronavirus Disease 2019 (COVID-19): A Randomized, Open-Label Clinical Trial. *Clinical Infectious Diseases* 2022; **75**(1): E432-E9. <https://doi.org/10.1093/cid/ciab962>
13. Ramatillah DL, Michael M, Khan K *et al.* Factors Contributing to Chronic Kidney Disease following COVID-19 Diagnosis in Pre-Vaccinated Hospitalized Patients. *Vaccines* 2023; **11**(2): 14. <https://doi.org/10.3390/vaccines11020433>
14. Leeyaphan J, Leeyaphan C, Suttha P *et al.* Healthcare Resource Utilization and Healthcare Costs of COVID-19 Patients in A Tertiary Care Public Hospital: A Retrospective Cohort Study in Thailand. *Journal of the Medical Association of Thailand* 2021; **104**(12): 1953-8. <https://doi.org/10.35755/jmedassocthai.2021.12.13109>
15. Ramatillah DL, Gan SH, Pratiwy I *et al.* Impact of cytokine storm on severity of COVID-19 disease in a private hospital in West Jakarta prior to vaccination. *PLoS One* 2022;**17**(11) <https://doi.org/10.1371/journal.pone.0262438>
16. Mohamad IN, Wong CKW, Chew CC *et al.* The landscape of antibiotic usage among COVID-19 patients in the early phase of pandemic: a Malaysian national perspective. *Journal of Pharmaceutical Policy and Practice* 2022; **15**(1) <https://doi.org/10.1186/s40545-022-00404-4>
17. Santoso P, Sung M, Hartantri Y *et al.* MDR Pathogens Organisms as Risk Factor of Mortality in Secondary Pulmonary Bacterial Infections Among COVID-19 Patients: Observational Studies in Two Referral Hospitals in West Java, Indonesia. *Int J Gen Med* 2022; **15**: 4741-51. <https://doi.org/10.2147/ijgm.S359959>
18. Dewi R, Kaswandani N, Karyanti MR *et al*. Mortality in children with positive SARS-CoV-2 polymerase chain reaction test: Lessons learned from a tertiary referral hospital in Indonesia. *International Journal of Infectious Diseases* 2021; **107**: 78-85. <https://doi.org/10.1016/j.ijid.2021.04.019>
19. Wayan Sumardika I, Cokro F, Wayan Suranadi I *et al.* Multidrug-resistant organism infections correlate with increased mortality in COVID-19 patients: A retrospective, observational cohort study. *Bali Journal of Anesthesiology* 2022; **6**(4): 231-4. <https://doi.org/10.4103/bjoa.bjoa_181_22>
20. Ong CCH, Farhanah S, Linn KZ *et al.* Nosocomial infections among COVID-19 patients: an analysis of intensive care unit surveillance data. *Antimicrobial Resistance and Infection Control* 2021; **10**(1). <https://doi.org/10.1186/s13756-021-00988-7>
21. Lie KC, Shakinah S, Pasaribu A *et al.* Observational Study on Secondary Bacterial Infection and the Use of Antibiotics in COVID-19 Patients Treated in a Tertiary Referral Hospital. *Acta Med Indones* 2022; **54**(2): 161-9. <https://www.actamedindones.org/index.php/ijim/article/view/2075/pdf>.
22. Soria MLJB, Quiwa LQ, Calvario MKJS *et al.* The philippine coronavirus disease 2019 (Covid-19) profile study: Clinical profile and factors associated with mortality of hospitalized patients. *Phillippine Journal of Internal Medicine* 2021; **59**(1): 37-58. <https://drive.google.com/file/d/16IC0Ps3GyU31cE3Stq0zRK5aIXngaI4H/view>.
23. Lau CL, Periyasamy P, Saud MN *et al.* Plethora of Antibiotics Usage and Evaluation of Carbapenem Prescribing Pattern in Intensive Care Units: A Single-Center Experience of Malaysian Academic Hospital. *Antibiotics (Basel)* 2022; **11**(9). <https://doi.org/10.3390/antibiotics11091172>.
24. See YP, Young BE, Ang LW *et al.* Risk Factors for Development of Acute Kidney Injury in COVID-19 Patients: A Retrospective Observational Cohort Study. *Nephron* 2021; **145**(3): 256-64. <https://doi.org/10.1159/000514064>
25. Vee ST, Muhamad DB, Nordin NB *et al.* Clinical characteristics of severe acute respiratory syndrome coronavirus 2 (Sars-cov2) patients in hospital tengku ampuan afzan. *Medical Journal of Malaysia* 2020; **75**(5): 479-84.
26. Asmarawati TP, Rosyid AN, Suryantoro SD *et al.* The clinical impact of bacterial co-infection among moderate, severe and critically ill COVID-19 patients in the second referral hospital in Surabaya. *F1000Res* 2021; **10**: 113. <https://doi.org/10.12688/f1000research.31645.2>
27. Nguyen TT, Pham TN, Van TD *et al*. Genetic diversity of SARS-CoV-2 and clinical, epidemiological characteristics of COVID-19 patients in Hanoi, Vietnam. *PLoS One* 2020; **15**(11): e0242537. <https://doi.org/10.1371/journal.pone.0242537>
28. Wannacharoen E. The Study of the Relationship of Procalcitonin (PCT) Levels and C-Reactive Protein (CRP) Levels in Patients Diagnosed by a Doctor Who is Infected with COVID-19, Phra Nakhon Si Ayutthaya Hospital in 2021. *JPMAT* 2023; **13**(1): 217-30. <https://he01.tci-thaijo.org/index.php/JPMAT/article/view/261210>
29. Kawila R. Clinical effectiveness of remdesivir in the treatment of moderate to severe and critical COVID-19 Infections: a retrospective study. *J Nakornping Hosp* 2021;**12**(2):183-95. <https://he01.tci-thaijo.org/index.php/jnkp/article/view/253097>
